# Early depletion of gut microbiota shape oligodendrocyte response after traumatic brain injury

**DOI:** 10.1186/s12974-024-03158-9

**Published:** 2024-07-15

**Authors:** Kirill Shumilov, Allen Ni, Maria Garcia-Bonilla, Marta Celorrio, Stuart H. Friess

**Affiliations:** 1 Department of Neurosurgery, Virginia Commonwealth University, Richmond, VA USA; 2 Department of Pediatrics, Washington University in St. Louis School of Medicine, St. Louis, MO USA; 3 Division of Critical Care Medicine, Department of Pediatrics, Washington University in St. Louis School of Medicine and St. Louis Children’s Hospital, Campus Box 8028, 3rd Fl MPRB 660 S. Euclid Avenue, St. Louis, MO 63110 USA

**Keywords:** Traumatic brain injury, White matter injury, Gut microbiota, T cells, Oligodendrocytes, Neuroinflammation, Remyelination

## Abstract

White matter injury (WMI) is thought to be a major contributor to long-term cognitive dysfunctions after traumatic brain injury (TBI). This damage occurs partly due to apoptotic death of oligodendrocyte lineage cells (OLCs) after the injury, triggered directly by the trauma or in response to degenerating axons. Recent research suggests that the gut microbiota modulates the inflammatory response through the regulation of peripheral immune cell infiltration after TBI. Additionally, T-cells directly impact OLCs differentiation and proliferation. Therefore, we hypothesized that the gut microbiota plays a critical role in regulating the OLC response to WMI influencing T-cells differentiation and activation. Gut microbial depletion early after TBI chronically reduced re-myelination, acutely decreased OLCs proliferation, and was associated with increased myelin debris accumulation. Surprisingly, the absence of T-cells in gut microbiota depleted mice restored OLC proliferation and remyelination after TBI. OLCs co-cultured with T-cells derived from gut microbiota depleted mice resulted in impaired proliferation and increased expression of MHC-II compared with T cells from control-injured mice. Furthermore, MHC-II expression in OLCs appears to be linked to impaired proliferation under gut microbiota depletion and TBI conditions. Collectively our data indicates that depletion of the gut microbiota after TBI impaired remyelination, reduced OLCs proliferation with concomitantly increased OLC MHCII expression, and required the presence of T cells. This data suggests that T cells are an important mechanistic link by which the gut microbiota modulate the oligodendrocyte response and white matter recovery after TBI.

**Supplementary Information:**

The online version contains supplementary material available at 10.1186/s12974-024-03158-9.

## Introduction

In the United States, approximately 1.7 million people experience traumatic brain injury (TBI) each year, and over 5 million face TBI-related disabilities [1]. The overall health cost attributable to non-fatal TBI in the United States has been estimated to be over $40 billion per year [2]. Traumatic white matter injury (WMI) is thought to be a major contributor to long-term cognitive dysfunction in TBI survivors [3–5]. Oligodendrocytes, which provide metabolic support to axons and are the producers of myelin in the central nervous system (CNS), undergo apoptosis after TBI, triggered by direct injury or in response to axonal degeneration [6, 7]. Mature oligodendrocytes present in the brain at the time of injury have limited, if any, capability to contribute to remyelination, with the bulk of CNS remyelination attributed to new oligodendrocyte production by oligodendrocyte lineage cells (OLCs) [8–10]. OLC-mediated remyelination involves a three-phase process of proliferation, recruitment, and differentiation, which can be adversely impacted by inflammation after TBI [11, 12].

Research over the past few years has revealed that the gut microbiota influence neurogenesis, myelination and subsequently functional and behavior outcomes [13, 14]. This complex interplay is known as the gut-brain axis, a bidirectional communication that includes neuro-endocrine-immunological activity [15]. Early-life gut microbial depletion has been shown to have long-term effects on maturation of myelinating oligodendrocytes leading to altered cognition and anxiety-like behavior [16]. The gut microbiota has been shown to modulate the inflammatory response after acute CNS injury impacting injury severity and recovery [17–19]. Recently, we showed that gut microbiota depletion after TBI alters the innate and adaptive immune response [17]. The innate immune response plays an important role in myelin debris clearance which influences OLC proliferation and migration [20]. Activated T-cells have been found to have a trophic role by promoting OLC proliferation in vitro [21], while effector T-cells inhibit OLCs differentiation in an inflammatory demyelinating mouse model [22]. However, the possible T cell-OLC crosstalk and the mechanism of cell-cell communication after WMI hasn’t yet been elucidated.

Gut microbiota perturbations are common after TBI [22, 23], but their potential impact on remyelination after traumatic WMI is currently unknown. Therefore, we hypothesized that the gut microbiota plays a critical role regulating the OLC response to traumatic WMI influencing T-cells differentiation and activation. We evaluated whether an intact enteric microbiome early after TBI is critical for OLC proliferation and remyelination. We further investigated if the presence of T cells is required for gut microbial modulation of the OLC response and subsequent remyelination following TBI. Finally, using an in vitro model system we further explored OLC/T-cell interactions and the role of the gut microbiome.

## Materials and methods

### Animals

All procedures were approved by the Washington University Animal Studies Committee (Protocol 19–0864) and are consistent with the National Institutes of Health guidelines for the care and use of animals. Animals were housed 5/cage and had free access to water and food with a 12-hour light/dark cycle. C57BL/6J (RRID: IMSR_ORNL: C57BL/6J-A/A) and B6.129P2-Tcrb^tm1Mom^Tcrd^tm1Mom/J^J (TCRβ^−/−^TCRδ^−/−^) (RRID: IMSR_JAX:002122) 8 week-old male and female mice (Jackson Laboratory, Bar Harbor, ME) weighing 20–25 g (g) were used for in vivo studies. C57BL/6J male and female neonatal P2-P5 pups were used for in vitro studies.

### Controlled cortical impact with gut microbial depletion

Controlled cortical impact (CCI) was performed using a previously described protocol [17]. Mice were anesthetized with 5% isoflurane and maintained at 2% isoflurane throughout the procedure. Buprenorphine sustained-release (0.5 mg/kg) was administered subcutaneously, prior to scalp incision. Ear bars were positioned to secure the head within the stereotaxic frame (MyNeurolab, St. Louis, MO). A 5 mm craniectomy was performed using an electric drill, centered 2.7 mm lateral to the midline and 3 mm anterior to lambda. Animals were randomly assigned to either CCI or sham group after craniectomy using a computer-generated randomization algorithm. The electronic impactor (Leica Biosystems, Richmond, VA) equipped with a 3 mm tip was aligned with the craniectomy site using the following coordinates: 1.2 mm lateral to the midline and 1.5 mm anterior to lambda [17]. The impact was delivered at a depth of 2 mm with a velocity of 5 m/s and a dwell time of 100 ms. A loose-fitting 7 mm plastic cap was secured over the craniectomy site using Vetbond (3 M, St. Paul, MN). The skin incision was closed with interrupted sutures and treated with antibiotic ointment. Animals were then placed on a warming pad for recovery.

For gut microbiota depletion, broad-spectrum antibiotics were administered for 7 consecutive days in the drinking water consisting of 250 mg vancomycin, 500 mg neomycin sulfate, 500 mg ampicillin, 500 mg metronidazole (VNAM), and 10 g grape-flavored Kool-Aid (Kraft Heinz, IL, Chicago) in 500 mL of sterile-filtered water [25]. Control animals received only Kool-Aid in drinking water to reduce the bitter flavor of the antibiotics.

### Fecal microbiota transplantation

We performed two fecal microbiota transplantations (FMTs) of the gut microbiota from VNAM- or Kool-Aid-treated uninjured animals into germ-free (GF) mice as previously described [24]. Briefly at Day 17 and Day 10 prior to injury, GF mice received FMTs from animals with 7 days of VNAM or Kool-Aid treatment. For each extraction, fecal pellets were collected 7 days after initiation of VNAM or Kool-Aid treatment. The pellets were mixed with phosphate-buffered saline (PBS) and after 5 min, the sample was vortexed to break up the fecal matter and allowed to sit for ∼5 min for debris to settle. The supernatant was then removed with an uncut P1000 pipette tip into a sterile 5 mL tube to avoid material clogging the gavage needle. Mice were gavaged with a sample volume of 250 µL. Ten days after the last FMT, we performed CCI in all the mice and they remained in sealed cages in the animal facility for 7 days before being euthanized.

### CD3-cpecific monoclonal antibody injection

For in vivo depletion of CD3 expressing cells, mice were injected intraperitoneally (i.p.) with 200 µg of InVivoPlus anti-mouse CD3ε, clone 145-2C11 (1 µg/µl, Cat# BP0001-1, Bio X Cell, Lebanon, New Hampshire), starting 6 days before injury, followed by 100 µg injections every four days for 1 month, to ensure T-cells depletion. The control group received the same injection regime with InVivoPlus polyclonal Armenian hamster IgG (Cat# BP0091 Bio X Cell, Lebanon, New Hampshire).

### 5-bromo-2’-deoxyuridine (BrdU) treatment

To detect cell proliferation in the pericontusional corpus callosum (CC), animals received intraperitoneal injections of 5-bromo-2’-deoxyuridine (BrdU, Sigma-Aldrich, St. Louis, MO) 50 mg/kg i.p. daily for 4 consecutive days starting 3 days post-injury.

### Tissue processing

Mice were euthanized under isoflurane anesthesia, by transcardial perfusion with cold 0.3% heparin in PBS followed by 4% paraformaldehyde solution in PBS (PFA, Sigma-Aldrich, St. Louis, MO). Brains were post-fixed in 4% PFA for 24 h at 4 °C followed by equilibration in 30% sucrose for 48 h before sectioning. Using a freezing microtome, brains were cut, and four 50-µm thick cryosections with complete CC spaced 300 μm apart were used for the subsequent analysis.

### Myelin Black Gold II staining

Myelin Black Gold II (BGII, Histo-Chem, Jefferson, AR) staining was performed as previously described [26, 27]. Briefly, to visualize individual myelin fibers in the CC and quantify myelinated percent area, BGII staining was performed on four 50-µm thick slices spaced 300 μm apart with the most rostral slice being the first appearance of the dorsal hippocampus. Free-floating slices were rinsed 3 times with tris-buffered saline (TBS) for 5 min at room temperature (RT), and then incubated for 12 min at 60 °C in pre-warmed BGII solution (0.3% in 0.9% NaCl), followed by 2 washes in distilled water at RT. Next, slices were incubated in pre-heated sodium thiosulfate (1% in distilled water) at 60 °C for 3 min. After 3 washes in TBS, tissue was mounted on charged slides and dried overnight. Slides were dehydrated using a serial of graded alcohols (50%, 70%, 95%, and twice with 100%) and coverslipped with DPX (Sigma-Aldrich, St. Louis, MO). Images were generated using 20X objective with a Brightfield Zeiss Axio Scan Z1 microscope (Zeiss, White Plains, NY). Percent of myelinated area of the CC was quantified using ImageJ software [25]. The CC region of interest was defined as the area between the mid CC and the cingulum unless it was truncated by the injury.

### Fluorescence immunohistochemistry

Fluorescence immunohistochemical staining was performed on free-floating sections. Tissue was incubated with pre-heated HCl 1 N (Sigma-Aldrich) for 30 min at 45 °C to increase the antigen exposure for BrdU detection. After the three washes with PBS, 20% normal donkey serum, 3% bovine serum albumin, and 0.3% triton X-100 in PBS were used to block nonspecific staining for all antibodies. Sections with a thickness of 50 μm were stained with the primary antibodies (Table 1) at 4 °C overnight. The next day, antibody binding was detected by incubating sections with Alexa Fluor secondary antibody (Table 1) for 2 h in PBS with 0.3% triton X-100. Sections were mounted on glass slides in PBS, dried, and coverslipped with mounting medium for fluorescence with 4’,6-Diamidino-2-Phenylindole, (DAPI, Thermo Fisher Scientific, Waltham, MA).

**Table 1:** Overview of the antibodies for immunohistochemistry and flow cytometry used in this study

Antibody	Fluorophore	Clone	Species	Source	Product number
CD45	BV425	30-F11	Rat monoclonal	BioLegend	103,134
CD3ɛ	AF700	500-A2	Armenian Hamster monoclonal	BioLegend	100,320
CD4	BUV395	GK1.5	Rat monoclonal	BD Biosciences	565,974
CD8a	BV785	53 − 6.7	Mouse monoclonal	BioLegend	100,750
CD11b	BV510	M1/70	Rat monoclonal	BioLegend	101,263
MHC-II		AF6-120.1	Mouse monoclonal	eBioscience	46-5320-80
TNFa	APC	MP6-XT22	Mouse monoclonal	BioLegend	506,307
IL-4	PerCP-Cy5.5	11B11	Rat monoclonal	BioLegend	504,123
IL-17	PE-Cy7	TC11-18H10.1	Mouse monoclonal	BioLegend	206,921
Olig1			Rabbit polyclonal	Millipore	AB15620
BrdU		BU1/75	Rat monoclonal	Abcam	Ab6326
βAPP			Rat monoclonal	Abcam	Ab32136
dMBP			Rabbit polyclonal	Millipore	Ab5864
Secondary antibody	AF594		Donkey anti-rat	Thermo Fisher	A-21,209
Secondary antibody	AF647		Donkey anti-mouse	Thermo Fisher	A-31,571
Secondary antibody	AF488		Donkey anti-rabbit	Thermo Fisher	A-21,206
Secondary antibody			Biotinylated goat anti-rabbit	Vector Laboratories	BA-1000-1.5

### Quantitative fluorescent immunohistochemistry

Fluorescent images were obtained with a Zeiss Axio Imager Z2 with ApoTome 2 fluorescence microscope with a 20X objective. 20-µm z stacks with an interval of 1 μm were obtained of the ipsilateral CC. Quantification of OLC proliferation was performed by counting the number of cells that co-localized with nuclear and/or cytoplasmic Olig1 immunolabeling and BrdU staining in 4 slices spaced 300 μm apart by a blinded observer. Degraded myelin basic protein (dMBP) fluorescent immunostaining was performed on adjacent sections. Images were generated using 20X objective with a fluorescence slide scanner Zeiss Axio Scan 7 microscope (Zeiss, White Plains, NY). dMBP percent area of the CC was quantified using ImageJ software [25].

### Primary oligodendrocyte lineage cell culture

Primary OLC culture and isolation was performed as previously described with a modified protocol [26, 27]. Briefly, brains from postnatal day P2-5 mice pups were isolated under a dissection microscope. Then, the cortical tissue were dissociated with a digestion cocktail containing papain (1.5 mg/ml, Worthington, Lakewood, OH) in a tissue culture incubator at 37 °C and 5% CO_2_ for 55 min. After a centrifugation at 100 g for 7 min, cells were resuspended in proliferation medium with DEMEM (Gibco, Waltham, MA), glutamax (1%, Gibco, Waltham, MA), penicillin-streptomycin (1%, Gibco, Waltham, MA), and fetal bovine serum (10%, Gemini Bi-products, Sacramento, CA) and plated in T-25 culture flask pre-coated with poly-L-lysine (100 µg/ml, Sigma-Aldrich, St. Louis, MO), the dissociated cortical tissue where plated at a concentration of 2,5 brains/flask. The mixed glia culture was incubated at 37 °C and 5% CO_2_. When confluence reached 90–100% (day 3–4), flasks were shaken at 330 RPM overnight, to dislodge OLCs and microglia from astrocytes which strongly attach to the flask. The supernatant containing OLCs and microglia were collected and added to a 100 mm tissue culture dish. Petri dishes were incubated at 37 °C and 5% CO_2_ for 30 min, to allow microglia to adhere. Medium containing enriched OLCs was harvested and centrifuged at 300 g for 7 min. Cells were then seeded at a density of 1 × 10^4^ cells/well on a 96-well plate or 1 × 10^5^ cells/well on a 6 well plate coated with poly-L-lysine and grown at 37 °C and 5% CO_2_ for 2 days in OLC basal medium. OLC basal medium containing DEMEM/F12 (MilliporeSigma, Burlington, MA) was supplemented with N2 (1%, ThermoFisher), B27 (1%, ThermoFisher), penicillin-streptomycin (1%, Gibco, Waltham, MA), BSA (0.3%, Sigma-Aldrich, St Louis, MO), bEGF (10 ng/ml, PeproTech, Waltham, MA), FGF (10ng/ml, PeproTech) and PDGFaa (10 ng/ml PeproTech).

### T-cells isolation from spleen

Spleens were collected from injured animals treated with VNAM or Kool-Aid as described above. Splenocytes were obtained by mechanical shredding and filtered through a 70-µm cell strainer, and centrifuged at 500 g for 10 min. The resulting cell suspensions were incubated with red blood lysis buffer (Roche Diagnostics Gmbh, Mannheim, Germany) for 5 min at 4°C, centrifuged and filtered through 40-µm cell strainer. T-cells were then isolated following manufacturers protocol by negative selection using pan T-cell isolation kit II (Miltenyi biotec). This isolation kit is based on a cocktail of biotin-conjugated antibodies against CD11b, CD11c, CD19, CD49b, CD105, Anti-MHC-class II, and Ter-119. Cells were counted and co-cultured with OLCs at a density of 3 × 10^4^ cells/well on a 96-well plate or 3 × 10^5^ cells/well on a 6-well plate at 37 °C and 5% CO_2_ for 24 h.

### Immunocytochemistry

Cells were permeabilized using 0.3% TX-100 in PBS for 10 min. Then cells were incubated with HCl 1 N (Sigma-Aldrich, St. Louis, MO) for 30 min at 45 °C to increase the antigen exposure for BrdU detection. Non-specific antibody interactions were blocked using 20% NDS in PBS for 1 h. Primary antibodies (Table 1) were diluted in the same blocking solution and incubated overnight at 4 °C. The cells were washed with PBS and secondary antibodies (Table 1) diluted in PBS were incubated for 2 h. For nuclei detection, cells were incubated for 10 min with DAPI (1:5000, Life Technology, Carlsbad, CA) and stored at 4 °C in PBS. The immunofluorescent images were taken using Zeiss Celldiscover 7 (Zeiss, White Plains, NY) with 10x objective, 4 tiles per well were automatically scanned. We used ImageJ (NIH public software) particle analysis plugin with macro instructions [27] to quantify the number of cells and percent of area immunostained. To analyze the co-localization of BrdU and DAPI positive cells quantitative analysis of Mander’s coefficient were performed using the JACoP plugin for ImageJ.

### Flow cytometry analysis

After 24 h of co-culture with OPC, T-cells were stimulated for intracellular cytokines expression in vitro. Supernatant from the co-culture were collected centrifuged at 300 g for 1 min and resuspended in RPMI 1640 with glutamax, 10% FCS, 12.5 mM of Hepes (Gibco), 1% of Pen/Strep (Gibco, Waltham, MA), 50µM of B-mercaptoetanol (Sigma-Aldrich, St. Louis, MO) and 10 µg/ml of gentamycine (Sigma-Aldrich) with 100 ng/ml of phorbol 12-myristate 13-acetate (PMA), 1 µg/ml of ionomycine (Sigma-Aldrich) and 1x brefeldin (BioLegend, San Diego, CA) at 37 °C and 5% CO_2_ for 4 h. OPCs were detached from the surface of the culture plate by repetitive resuspensions in FACS buffer and collected in 1 ml tubes, followed by centrifugation at 300 g for 7 min. Next, cells were incubated for 5 min with Zombie NIR Dye (BioLegend, San Diego, CA). Then, cells were washed with FACS buffer, stained with their respective antibody mix (Table 1) for 30 min at RT, and analyzed on a BD LSRFortessa flow cytometer (BD Biosciences, Franklin Lakes, NJ) using the Software v10.6.1 (BD Biosciences, Franklin Lakes, NJ). T-cells were defined as CD45^high^CD11b^−^CD3^+^.

### Intracellular cytokine staining and analysis

Cells were incubated for 5 min at RT with Zombie NIR Dye (BioLegend, San Diego, CA) to assess their viability. The Zombie NIR Dye was quenched, and cells were washed with cytometry buffer and blocked with FcR blocking reagent (1:50, Miltenyi Biotec, Bergisch Gladbach, Germany). Then, the samples were washed with cytometry buffer, stained with antibodies (Table 1) for 15 min at RT, Microglial cells were defined as CD45lowCD11b + and T cells as CD45hiCD11b − CD3+. For the intracellular staining, cells were first stained with surface markers as indicated above, stimulated, fixed and permeabilized by using FoxP3/transcription factor staining buffer set (eBiosciences) following the manufacturer’s instructions. Briefly, cells were stimulated for 4 h with 0.2 µg/mL phorbol 12-myristate 13-acetate (PMA)/ 2 µg/mL ionomycin/ 1x brefeldin A to characterize T cell subset changes associated with GMD after TBI. Next cells were fixed for 7 min at 4 °C, washed, permeabilized and stained with intracellular markers (Table 1) for 30 min at 4 °C. We measured the percentages of IL-17 + T cells. Then, cells were washed and analyzed on a BD LSRFortessa flow cytometer (BD Biosciences, Franklin Lakes, NJ) using the Software v10.6.1 (BD Biosciences, Franklin Lakes, NJ). Fluorescence minus one (FMO) and isotype control antibodies were used as negative controls for each marker.

### Statistical analysis

Blinding of investigators to experimental groups was maintained until data were fully analyzed. Data were assessed for normal distribution with the Shapiro-Wilk test and expressed as mean ± SEM. Two-tailed Student’s t-test was used when comparing two conditions. For more than two conditions, ANOVA and Tukey’s multiple comparison post-hoc test were employed. All analysis was performed with GraphPad Prism v10.1.0 (GraphPad software. Boston, MA).

## Results

### Gut microbiota depletion impedes white matter repair three months after TBI

**Fig. 1:**
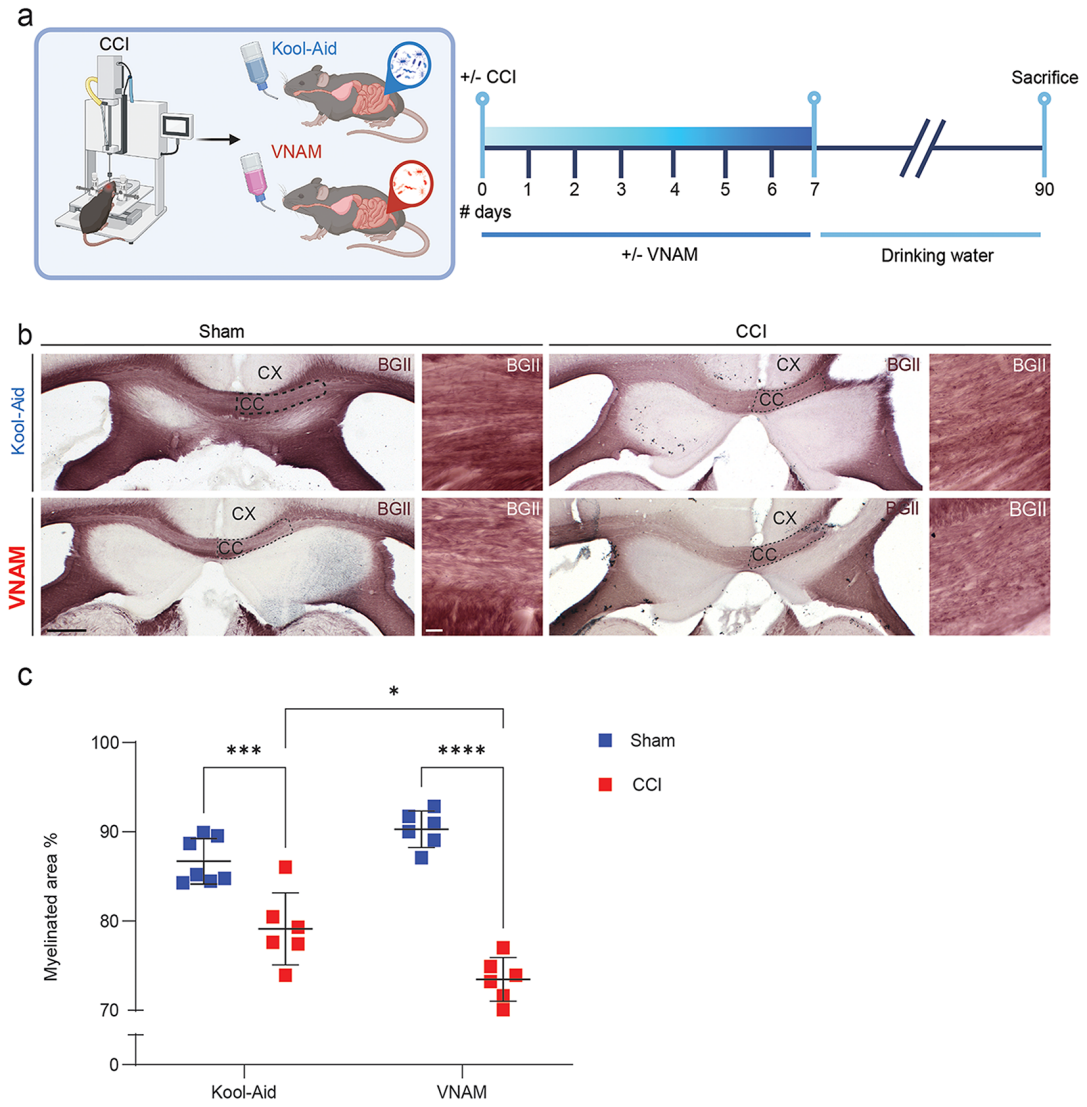
**Gut microbial depletion impairs remyelination after TBI**. **a** Experimental design: one week of VNAM treatment after TBI; animals were sacrificed 3 months after injury. **b** Representative images of the CC (black dashed lines) areas stained with BGII. **c** Quantification of percentage of the myelinated area of CC interaction of injury and gut microbiome F_(1,21)_ = 16.29 *p* = 0.0006 interaction of VNAM and CCI. Mean values are plotted ± SEM. ***p = 0.0006; ****p < 0.0001. Two-way ANOVA followed by Tukey multiple comparison post hoc test was used to determine statistical differences; *n* = 6–7 mice per group. Scale bar = 200 μm and 50 μm in the inserts. Abbreviations: CC: corpus callosum; CCI: controlled cortical impact; CX: cortex; BGII: myelin black gold II; VNAM: vancomycin, neomycin-sulfate, ampicillin, and metronidazole

Our previous work has demonstrated that gut microbial depletion for 1 week significantly impacts neuroinflammation and fear memory 3 months after TBI [17]. Since, chronic white matter degeneration is influenced by prolongated neuroinflammation [28], we decided to investigate the impact of gut microbiota depletion on white matter remyelination 3 months after TBI. Gut microbiota was depleted by administering broad-spectrum antibiotics orally to mice immediately after CCI for 1 week (Fig. 1a). Three months post-TBI, white matter remyelination was assessed by staining the peri-contusional CC with BGII and measuring the percentage of myelinated area adjacent to the lesion site (Fig. 1b and c). Our findings revealed that early gut microbiota depletion after injury significantly reduced the percentage of myelinated area in the CC compared with Kool-Aid-treated animals 3 months post-TBI (Fig. 1c). However, the underlying mechanisms by which gut microbiota influences post-TBI recovery remain to be elucidated. Therefore, we next performed a more in-depth analysis of gut microbiota depletion impact on acute WMI.

### Depletion of gut microbiota decreases oligodendrocyte lineage cell proliferation and increases myelin debris one week after TBI

**Fig. 2:**
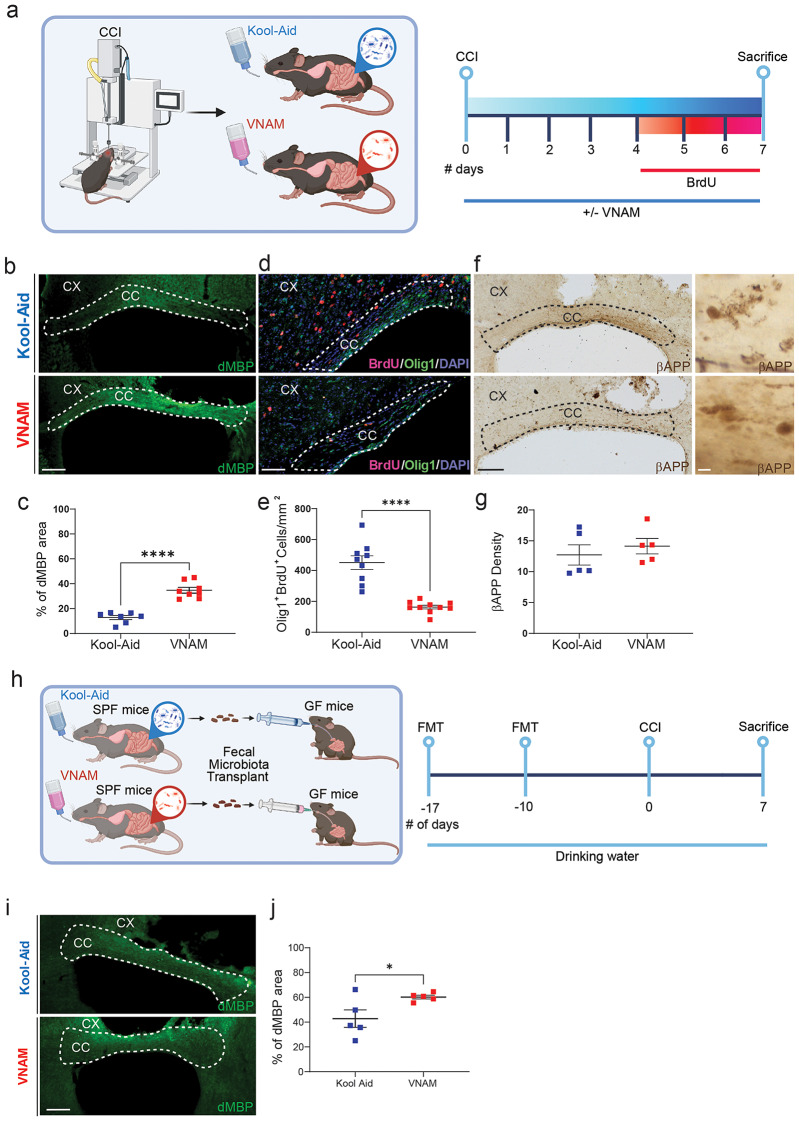
**Gut microbial depletion reduces oligodendrocyte lineage cell proliferation after TBI**. **a** Experimental design: one week of VNAM treatment after TBI; animals were sacrificed one week after injury. Representative images of CC (white dashed lines) stained with **b** dMBP, **c** Olig1^+^/BrdU^+^, and **d** bAPP. **e** Quantification of percentage of the dMBP-stained area of the CC. **f** Quantification of Olig1^+^/BrdU^+^ cells in the ipsilateral CC. **g** Quantification of βAPP axonal swelling density of the CC. **h** Experimental design: Germ free (GF) mice were gavaged with two fecal microbiota transplants (FMT) of the gut microbiota from mice treated with VNAM or Kool-Aid uninjured animals on Day − 7 and day − 17 prior to injury. **i** Representative fluorescent images of CC (white dashed lines) stained with dMBP. **j** Quantification of percentage of the dMBP-stained area of the CC. Mean values are plotted ± SEM. *p < 0.05. Unpaired t tests was used to determine statistical differences; *n* = 5–9 mice per group. Scale bar = 200 μm and 50 μm in the inserts. Abbreviations: CC: corpus callosum; CCI: controlled cortical impact; CX: cortex; dMBP: degraded myelin basic protein; FMT: fecal matter transplant; GF: germ free; SPF: specific pathogens free; VNAM: vancomycin, neomycin-sulfate, ampicillin, and metronidazole

Next, we wanted to further explore the effect of gut microbiota depletion on acute WMI and remyelination 7-day post-TBI (Fig. 2a). One critical inhibitor of remyelination and oligodendrocytes proliferation is myelin debris accumulation [29]. After WMI, myelin debris is accumulated specially in CC due to glial dysfunction and can be associated with axon regeneration impairment and neuroinflammation [30]. We found an increase in myelin debris accumulation in the CC of VNAM-treated injured mice compared with injured controls (Fig. 2b-c). Additionally, gut microbiota depletion suppressed OLC proliferation, a crucial step in remyelination, as indicated by a significant reduction in BrdU/Olig1 double-positive cells (Fig. 2d-e). The density of β-APP swelling, indicative of axonal injury, did not differ between VNAM and Kool-Aid treated mice one week after TBI (Fig. 2f-g). Furthermore, to determine if the impact of gut microbiota depletion on the white matter remyelination after TBI was mediated directly by antibiotics or indirectly via modulation of the gut microbiota, we utilized a fecal microbiota transplantation (FMT) approach (Fig. 2h). Consistent with the results described above, GF mice that received FMT from VNAM-treated animals exhibited significantly higher percent area of myelin debris accumulation compared to those receiving Kool-Aid FMT (Fig. 2i-j). Taken together, these results support our hypothesis that the gut microbiota plays a pivotal role in modulating post-traumatic myelin debris clearance and OLC proliferation.

### Pharmacological depletion or genetic deletion of T-cells rescues OLC proliferation and remyelination impaired by gut microbial depletion

**Fig. 3:**
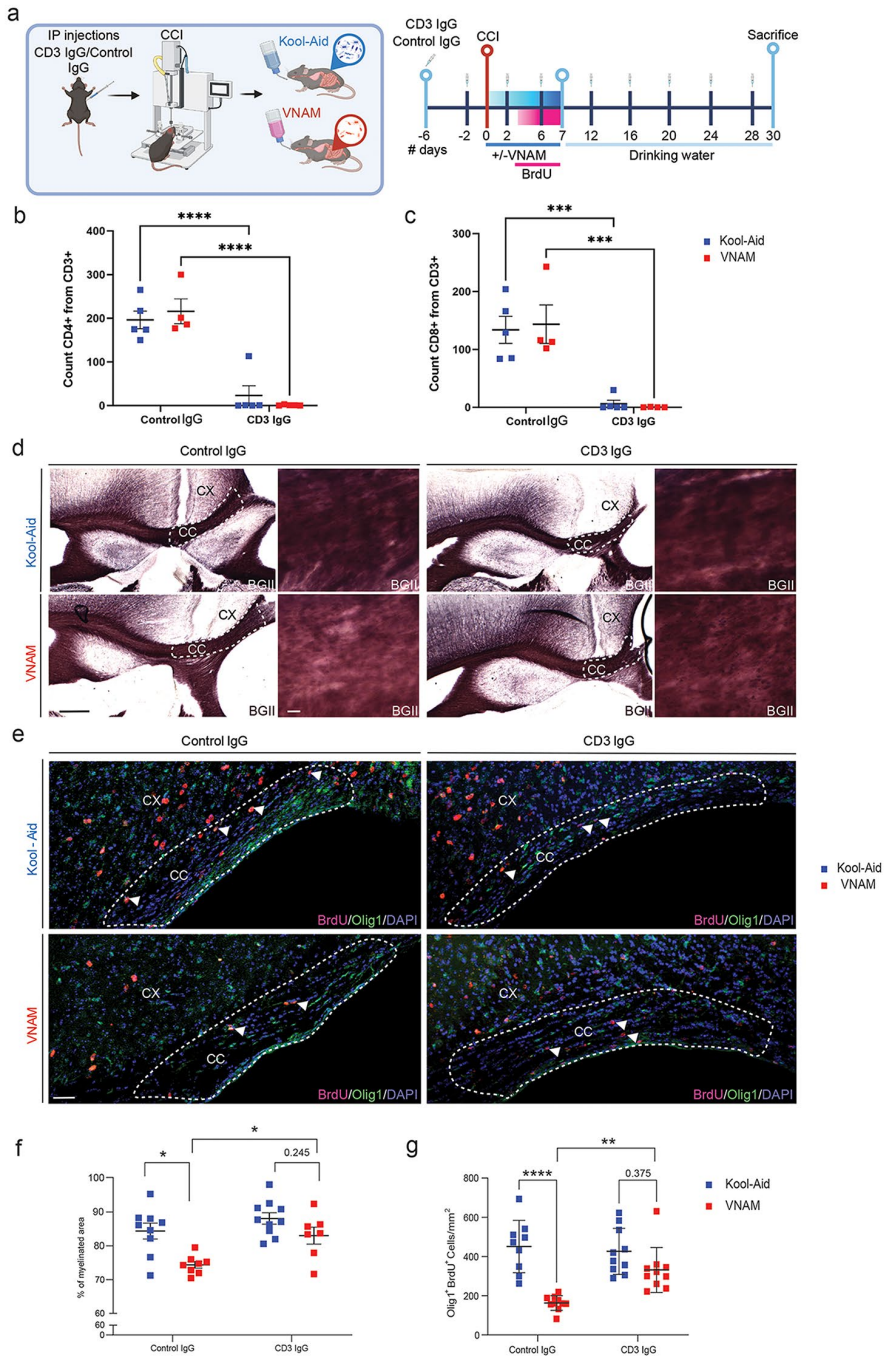
**Pharmacological depletion and genetical deletion of T-cells restore OLCs proliferation after TBI and gut microbial depletion**. **a** Experimental design: six days before injury animals received the first injection of CD3 IgG/control IgG then every four days; one week of VNAM treatment after TBI; animals were sacrificed one month after injury. Quantification of cell absolute numbers in the blood for **b** CD4 T-cells (CD11b^−^CD3^+^CD4^+^) and **c** CD8 T-cells (CD11b^−^CD3^+^CD8^+^). **d** Representative images of the CC (black dashed lines) areas stained with BGII. **e** Representative fluorescent images of CC (white dashed lines) stained with Olig1/BrdU. **f** Quantification of percentage of the myelinated area of CC F_(1,30)_ = 1.572 *p* = 0.2195 interaction of VNAM and CD3 depletion. **g** Quantification of Olig1^+^/BrdU^+^ proliferative cell density of the CC F_(1,35)_ = 7.986 *p* = 0.0077 interaction of VNAM and CD3 depletion. Mean values are plotted ± SEM. ****p < 0.0001; ***p < 0.0006; **p < 0.005. Two-way ANOVA followed by Tukey multiple comparison post hoc test was used to determine statistical differences; *n* = 6–7 mice per group. Scale bar = 200 μm and 50 μm in the inserts. Abbreviations: CC: corpus callosum; CCI: controlled cortical impact; CX: cortex; BGII: myelin black gold II; VNAM: vancomycin, neomycin-sulfate, ampicillin, and metronidazole

**Fig. 4:**
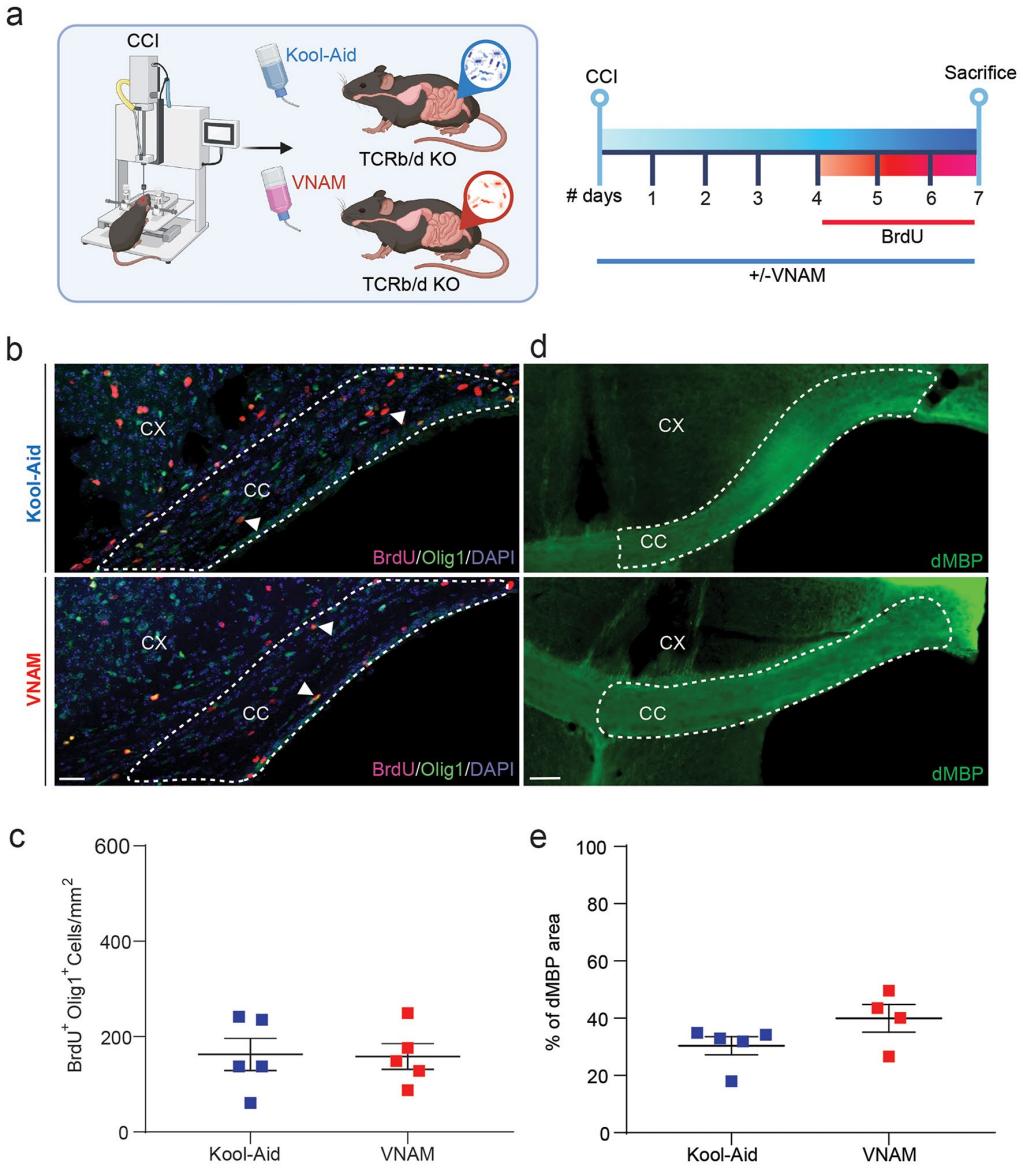
**Gut microbial depletion after TBI does not impact OLC proliferation and dMBP accumulation in TCRβ**^**-/-**^**TCRδ**^**-/-**^**mice. a** Experimental design: one week of VNAM treatment after TBI; animals were sacrificed one week after injury. **b** Representative fluorescent images of CC (white dashed lines) stained with Olig1 and BrdU. **c** Quantification of Olig1^+^/BrdU^+^ proliferative cell density of the CC. **d** Representative images of CC (white dashed lines) stained with dMBP. **e** Quantification of percentage of the myelinated area of CC. Mean values are plotted ± SEM. Unpaired t tests was used to determine statistical differences; *n* = 4–5 mice per group. Scale bar = 200 μm. Abbreviations: CC: corpus callosum; CCI: controlled cortical impact; CX: cortex; dMBP: degraded myelin basic protein; VNAM: vancomycin, neomycin-sulfate, ampicillin, and metronidazole

Previously, we have demonstrated that gut microbiota depletion after TBI impairs hippocampal neurogenesis, promotes pro-inflammatory microglia phenotype and surprisingly reduced T cell infiltration into the brain up to one month after injury [17] but then normalizes by 3 months after injury (Supplemental Fig. 1). Building upon these findings, we wanted to further investigate the role of the T-cells in gut-brain communication in the context of WMI. To achieve the depletion of T-cells, we employed pharmacological treatment with anti-CD3 IgG for one month, to be able to address changes in myelin density (Fig. 3a). Peripheral blood analysis following T-cell depletion revealed a nearly complete absence of CD4^+^ (Fig. 3b) and CD8^+^ (Fig. 3c) lymphocytes. Surprisingly, the percent of myelinated area (Fig. 3d and f) and impaired OLC proliferation (Fig. 3e and g), effect of gut microbial disruption, was restored after the depletion of T-cells compared to VNAM-treated mice with control IgG injections. Furthermore, TCRβ^−/−^TCRδ^−/−^ mice (absence of alpha beta T-cell receptor and any gamma delta T-cell receptor) exposed to VNAM for one week (Fig. 4a) showed similar findings. The density of oligodendrocyte lineage proliferative cells (Fig. 4b-c), and myelin debris accumulation (Fig. 4d-e) remained unaffected by microbial depletion in TCRβ^−/−^TCRδ^−/−^ mice. Taken together, these findings suggest that T-cells have a crucial role in gut-brain communication and the modulation of remyelination following traumatic WMI.

### In vitro co-culture of T-cells derived from injured mice with gut microbiota depletion reduced proliferation of OLCs

**Fig. 5:**
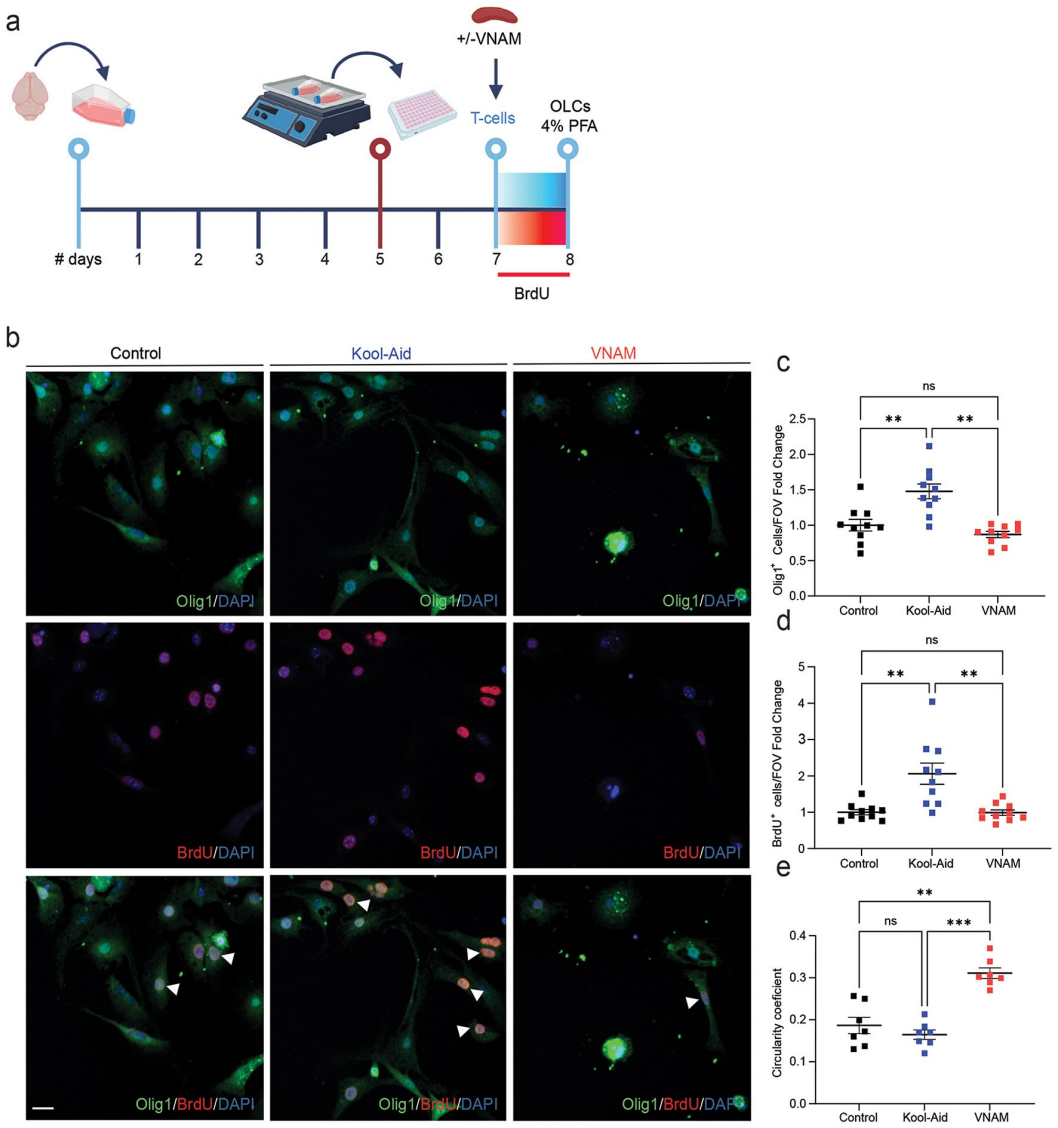
**Co-culture of T-cells derived from injured animals with gut microbiota depletion impairs OLCs proliferation**in vitro. **a** Experimental design: Oligodendrocyte lineage cells were isolated form P5 mice brains and cultured for five days. **b** Representative fluorescent images stained Olig1 and BrdU. **c** Quantification of Olig1 cell density F_(1.99,17.99)_ = 14.85 *p* = 0.0002 VNAM treatment interaction. **d** Quantification of BrdU proliferative cell density F_(1.215,10.95)_ = 14.32 *p* = 0.0022 VNAM treatment interaction. **e** Quantification of cell circularity coefficient F_(1.582,9.495)_ = 22.96 *p* = 0.0004 VNAM treatment interaction. Mean values are plotted ± SEM. ***p < 0.0005; **p < 0.005. One-way ANOVA followed by Tukey multiple comparison post hoc test was used to determine statistical differences; *n* = 10 wells per group. Scale bar = 20 μm. Abbreviations: OLCs: oligodendrocyte lineage cells; PFA: paraformaldehyde; VNAM: vancomycin, neomycin-sulfate, ampicillin, and metronidazole

OLCs are susceptible to damage from inflammatory environments triggered by T-cell cytokine production (Larochelle et al., 2021). This exposure leads to a significant shift in their gene expression profile (Falcao et al., 2018). Cytokines, such as IL17 further modulate OLC functions, leading to apoptotic death of oligodendrocytes [31]. Since, the absence of T-cells mitigated the impact of gut microbiota depletion on traumatic WMI repair, we decided to investigate the mechanism of interaction between OLCs and T-cells in vitro (Fig. 5a). T-cells isolated from spleens of injured animals with and without microbiota depletion 7 days after injury, were co-cultured with OLCs. We found decreased Olig1^+^ (Fig. 5c), and BrdU^+^ (Fig. 5d) cell density when OLCs were co-cultured with T-cells from injured mice with microbiota depletion. Analysis using Mander’s coefficient revealed co-localization of 96% of BrdU staining with Olig1 (0.9606 ± 0.02), indicating that the vast majority of proliferating cells were OLCs. Additionally, morphological analysis of OLCs showed an increase of circularity coefficient, indicative of a decreased maturation [32], when T-cells derived from injured animals with gut microbiota depletion were added to the medium (Fig. 5e). To further explore this cellular interaction, medium from T-cell and OLCs culture where exchanged (Sup. Figure 1a), however, no changes of Olig1 or BrdU density was detected after 24 h of culture (Sup. Figure 1b-c).

**Fig. 6:**
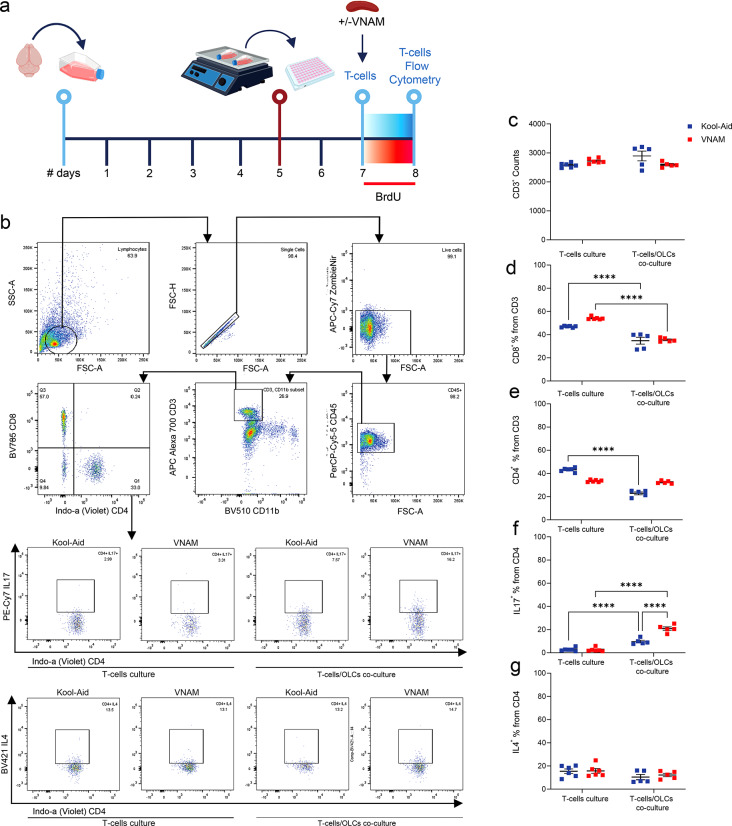
**Co-culture of OLCs increased IL17 CD4**^**+**^**T-cells derived from injured animals with gut microbiota depletion**. **a** Experimental design: cells were isolated form P5 mice brains and cultured for five days; after overnight shaking supernatant cells were co-cultured in plates with T-cells for 24 h. **b** Gating strategy. **c** Quantification of CD3^+^ cells. **d** Quantification of the % of CD8^+^ cells from CD3^+^ T-cells F_(1,18)_ = 5.24. **e** Quantification of the % of CD4^+^ cells from CD3^+^ T-cells F_(1,18)_ = 163.8. **f** Quantification of the % of IL17^+^ cells from CD4^+^ T-cells F_(1,18)_ = 34.76. **g** Quantification of the % of IL4^+^ cells from CD4^+^ T-cells. Mean values are plotted ± SEM. ****p < 0.0001. Two-way ANOVA followed by Tukey multiple comparison post hoc test was used to determine statistical differences; *n* = 6–9 wells per group. Abbreviations: VNAM: vancomycin, neomycin-sulfate, ampicillin, and metronidazole

Next, we characterized the T-cell differentiation from the in vitro co-culture using flow cytometry (Fig. 6a). No significant differences were observed in the total number of CD3^+^ lymphocytes (Fig. 6b). However, the percentage of CD8^+^ (Fig. 6c) were significantly lower in both co-culture groups, and CD4^+^ (Fig. 6e) showed a decrease only in the Kool-Aid group. The percentage of CD4^+^ T-cells expressing IL17 (Fig. 6f) cytokines was significantly higher in lymphocytes derived from spleens of injured mice with depleted gut microbiota compared with injured controls. No significative changes were detected when IL4 (Fig. 6g) expression was analyzed. Furthermore, when OPCs medium was added to T-cells culture a similar increase IL17 (Sup. Figure 1 g) was detected in T cells derived from gut microbiota depleted mice. Collectively, our findings provide evidence that differentiation of T-cells towards more pro-inflammatory phenotype could impair white matter repair after TBI through the modulation of OLC proliferation.

**Fig. 7:**
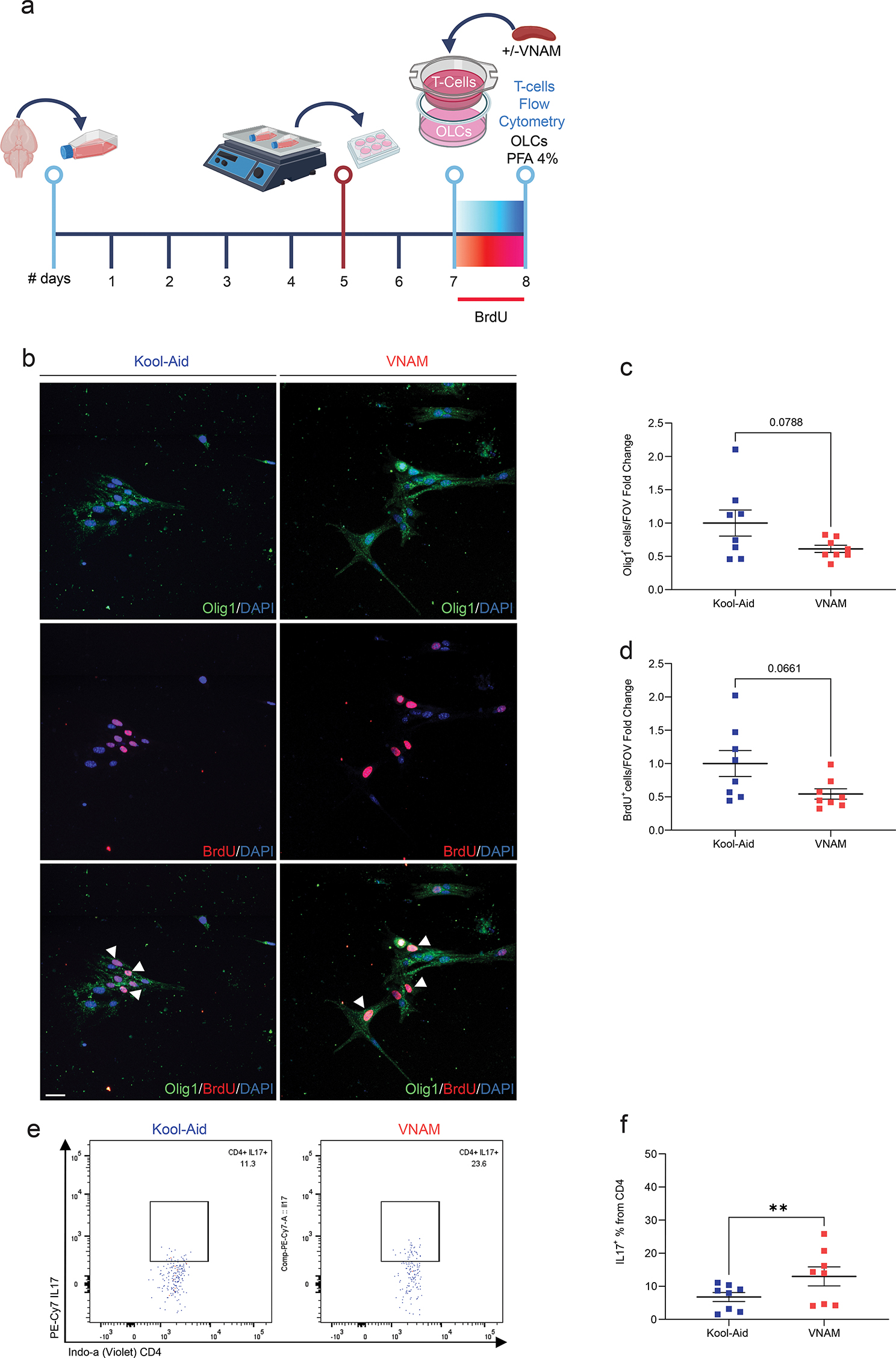
**Trans-well co-culture of T-cells derived from injured and microbiota depleted animals with OLCs partially reduced OLCs proliferation and increased CD4**^**+**^**IL17 expression**. **a** Experimental design: cells were isolated form P5 mice brains and cultured for five days; after overnight shaking supernatant cells were cultured in plates with trans-well co-cultured T-cells for 24 h. **b** Representative fluorescent images stained Olig1/BrdU cells. **c** Quantification of Olig1 cell density. **d** Quantification of BrdU proliferative cell density. **e-f** Gating and quantification of the % of IL17^+^ cells from CD4^+^ T-cells. Mean values are plotted ± SEM. ***p < 0.0003 **p < 0.066; *p < 0.046. Unpaired t test was used to determine statistical differences; *n* = 7 wells per group. Scale bar = 20 μm. Abbreviations: OLCs: oligodendrocyte lineage cells; PFA: paraformaldehyde; VNAM: vancomycin, neomycin-sulfate, ampicillin, and metronidazole

To further elucidate the nature of T-cell and OLC interactions in vitro, we employed a trans-well co-culture system (Fig. 7a). The permeable barrier allowed media exchange while preventing direct cell contact. Interestingly, Olig1^+^ (Fig. 7c), and BrdU^+^ (Fig. 7d) presented a trend towards a decreased OLCs density. However, CD4^+^ lymphocytes derived from spleens of VNAM treated and injured animals exhibited significantly elevated expression of IL17 (Fig. 7f) cytokine compared to Kool-Aid controls. This finding suggests that, while modulation of OLC proliferation could have a greater impact when contact with T-cells is direct, CD4^+^ lymphocytes differentiation occurs indirectly through extracellular signaling. Our findings provide additional evidences of the immunomodulatory role of OLCs, highlighting their complex bidirectional interplay with T-cells.

### Gut microbiota depletion increase T-cell-induced MHCII expression in OLCs after TBI

**Fig. 8:**
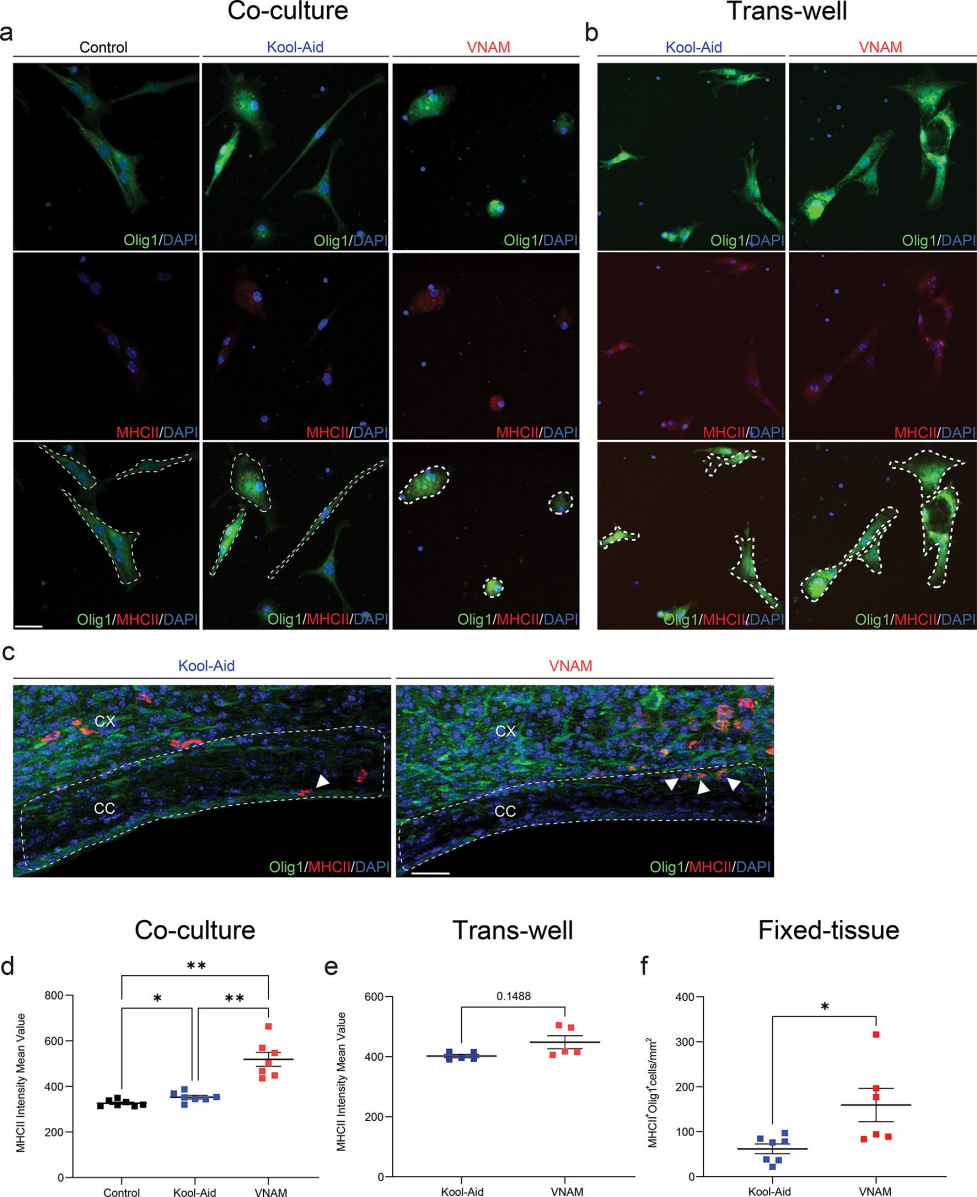
**Gut microbial depletion after TBI increase MHCII expression in OLCs through direct T-cell contact**. **a** Representative fluorescent images of Olig1^+^/MHCII^+^ staining of OLCs 24 h after T-cell co-culture. **b** Representative fluorescent images of Olig1/MHCII staining of OLCs 24 h after T-cell trans-well co-culture. **c** Representative fluorescent images of CC (white dashed lines) stained with Olig1/MHCII. **d** Quantification of MHCII intensity mean value of OLCs 24 h after T-cell co-culture F_(1.078,6.469)_ = 22.95 *p* = 0.0014 VNAM treatment interaction. **e** Quantification of MHCII intensity mean value of OLCs 24 h after T-cell trans-well co-culture. **f** Quantification of Olig1^+^/MHCII^+^ cell density of the CC. Mean values are plotted ± SEM. ***p < 0.0003 **p < 0.0026; *p < 0.0449 One-way ANOVA followed by Tukey multiple comparison post hoc test and Unpaired t tests was used to determine statistical differences; *n* = 7 wells per group in d; *n* = 5 wells per group in e; *n* = 6–7 mice per group in f. Scale bar = 20 μm in a and b. Scale bar = 200 μm in c. Abbreviations: CC: corpus callosum; CX: cortex; VNAM: vancomycin, neomycin-sulfate, ampicillin, and metronidazole

There is a growing body of evidence suggesting that OLCs can express immunomodulatory factors such as cytokines/chemokines and their receptors [33, 34] et al., 2014). T-cells/OLCs bidirectional communication was described in a previous study, where OLCs increased expression of immunoprotective genes suggesting a potential mechanism of immune functions in the context of multiple sclerosis (MS) disease [35]. To further explore the mechanism of bidirectional communication between T-cells and OLCs, we decided to analyze major histocompatibility complex II (MHC-II) expression. The presence of genes associated with MHC class I and II was found in OLCs in response to interferon exposure [35, 36]. OLCs MHC-II intensity values was analyzed in vitro, co-cultured with direct T-cells contact (Fig. 8a), and with trans-wells cell culture (Fig. 8b). Additionally, Olig1^+^/MHC-II^+^ cell density was analyzed in the peri-contusional CC (Fig. 8c). We found that OLCs exposed to T-cell derived from microbiota depleted animals had a significantly higher MHC-II intensity mean value compared with control (Fig. 8d). Surprisingly, this difference vanished under in vitro trans-well co-culture conditions (Fig. 8e), suggesting that cell-to-cell contact might be necessary for T-cell-mediated MHC-II expression on OLCs. Furthermore, Olig1 and MHC-II co-localization significantly increased in peri-contusional CC of injured and VNAM-exposed animals compared with injured controls (Fig. 8f), indicating potential activation of immunomodulatory functions in OLCs. This data supports the potential immunomodulatory functions of OLCs under gut-microbial depleted context and the pivotal role of T cells in gut-brain communication.

## Discussion

This report provides evidence that the gut-brain axis influences white matter remyelination after TBI. Depletion of the gut microbiota after TBI impaired OLC proliferation and resulted in long-term reductions in white matter remyelination. Furthermore, our data suggests that T-cells play an important mechanistic link in gut-brain communication in regards to OLC proliferation and remyelination. Consistent with observations of MS related disease, where MHC-II expressing OLCs could activate effector T-cells [35], we show that T-cells from a gut microbial depleted host could differentiate into IL17 CD4^+^ in presence of OLCs in vitro. While MHC-II expression in OLCs appears to be linked to impaired proliferation. Collectively, our findings suggest that oligodendrocytes are not passive in the neuroinflammatory and degenerative environment caused by brain trauma but instead could exert an active role in the modulation of immune response.

Extensive research has established the microbiome’s influence on inflammatory responses and immunology [37–39]. Notably, its role in shaping brain development and regulating central nervous system (CNS) functions is becoming increasingly evident [40]. Disruption of gut microbial composition and diversity has been linked to various neurodevelopmental disorders, including autism spectrum disorders, depression, and schizophrenia [38, 41, 42]. Furthermore, the gut microbiome has the potential to modulate white matter structural integrity in a diet-dependent manner [43]. Our investigation into TBI and gut microbial depletion reveals a profound impact on WMI recovery. We observed impaired white matter remyelination, characterized by decreased OLC proliferation and myelin debris accumulation, following TBI in animals with depleted microbiota. This correlation was further demonstrated through microbial transplantation in GF mice, showing a direct impact of the gut microbiota on WMI. This evidence emphasizes the gut microbiota’s potential as a modulator of post-TBI recovery.

TBI triggers complex immune responses, including the recruitment of lymphocytes to the injured site. While T-cell infiltration was traditionally linked to worsened outcomes and exacerbated brain damage [44], other research suggests that specific T-cell populations might actually play a protective role in brain injury recovery [45, 46]. Gut microbiota are known to regulate the immune cell response after TBI. In our previous work injured mice with gut microbial depletion were found to have altered microglial morphology and increased neurodegeneration associated with impaired T-cell infiltration [17]. In this manuscript, we found that in the absence of T-cells, gut-microbiota depletion had a significantly reduced impact on myelin repair and OLC proliferation following injury. This effect was observed both in the pharmacological deletion of CD3^+^ lymphocytes and in genetic absence of T-cells in TCRβ^-/-^TCRδ^-/-^ mice. These findings provide evidence for a critical role of T-cells in gut-brain communication in the setting of TBI. Further elucidating the mechanistic and regulatory interactions between the gut microbiota, the immune response, and the brain in the setting of TBI can provide foundational knowledge for the development novel therapeutic strategies to enhance white matter repair.

While the role of resident immune cells in the CNS is well-established, the involvement of non-immune glial cells like astrocytes and OLCs in neuroinflammation has only recently emerged as a critical area of research. In the context of neurological disorders, OLCs can transition to disease-specific cell states [47]. Disease-specific OLCs are characterized by the expression of immune specific genes allowing the direct cross-tale between immune cell and therefore modulating immune response [35]. Furthermore, defective perivascular migration of OLCs not only impairs their recruitment to the lesion site but can also disrupt the blood brain barrier, making it more permeable to infiltrating CD3^+^ lymphocytes [48]. To further understand the role of T-cells in the modulation of OLCs we performed an in vitro co-culture with both cell types. We found that OLCs exposed to T-cells from gut-microbiota-depleted and injured mice exhibited stunted proliferation. This suppression was partially mediated by direct cell contact. Analysis of T-cells population revealed an increase in CD4^+^ lymphocytes expressing IL17 in gut-microbiota-depleted mice. This finding aligns with existing evidence suggesting that immune cells modulate OLC proliferation through IL17 [31, 49]. Furthermore, CD4^+^ T-cells of the Th1 and Th17 lineage play a pivotal role in MS perpetuation and establishment [50, 51]. Emerging evidences point to a role of Th17 cells in a wide variety of cognitive, neurovascular, and neurodegenerative diseases [52]. However, additional research is required to uncover the precise mechanisms behind the gut microbial influence on white matter repair after injury.

The overall immunomodulatory role of OLCs in CNS diseases and disorders still needs to be investigated. Emerging evidence suggests that OLCs could modulate immune cells activation through the increased expression of gene modules associated with interferon response and MHC class I and II [35, 36]. These studies established a novel role of OLCs in antigen presentation in vivo [35, 36, 53]. This novel OLCs function was further confirmed by single-nuclei RNA sequencing analysis in cortical gray matter and subcortical white matter [54]. This study revealed an increased expression of MHC genes as signature of stressed oligodendrocyte in MS lesions [54]. We then wondered whether T-cells isolated from injured and gut microbiota depleted animals could influence MHC class II expression in vitro. Our data revealed that MHC-II was upregulated in OLCs in cell contact dependent manner. Furthermore, increased colocalization of MHC-II and Olig1 was observed in the peri-contusional CC after TBI with gut microbial depletion. These findings suggest a potential mechanism by which T-cells might directly interact with OLCs through MHC-II, modifying their proliferation and amplifying their immunomodulatory role. Further research is crucial to determine whether OLCs immunomodulatory functions are prominent or merely limited to a fine-tuning effect. Our in vivo and in vitro data implicate T helper cells (CD4^+^) as the critical T cell subset interacting with OLCs. Future studies with specific antibody depletion of CD4^+^ T cells are planned.

There are several limitations in our investigation. Our in vivo studies included young adult mice, but the response to injury/recovery and gut dysbiosis may be influenced by sex and age [55, 56]. Furthermore, gut dysbiosis and systemic inflammation can be influenced directly by age-related dysregulation of bile acid homeostasis [57, 58]. Future research should prioritize exploring this complex interplay in the context of white matter repair. The level of antibiotics remaining in the feces of SPF mice, transplanted to GF mice, wasn’t analyzed. It is possible that the GF mice had some exposure to VNAM during FMT. However, the antibiotics used are not systemically absorbed (except for metronidazole) and the second FMT was, performed 10 days before injury. We used a focal injury model which limited our ability to evaluate other white matter regions affect in human TBI. Future studies using animal models of diffuse TBI are warranted. Another limitation of our study is the absence of sham control in the analysis of OLCs proliferation, myelin debris accumulation and axonal swelling in both WT and TCRβ^-/-^TCRδ^-/-^ mice. Nevertheless, the proliferation of OLCs in the CC of adult uninjured mice are inherently rare under homeostatic conditions as well as the accumulation of myelin debris and axonal swelling. The absence of in vitro model of myelination and OLCs differentiation could be considered as a limitation to our study. Previous studies have identified accelerated remyelination in brain slice cultures with regulatory T-cells, that directly promoted OPCs differentiation [51, 59]. Co-staining with a marker of mature oligodendrocytes, such as proteolipid protein, or OPC specific marker PDGFr-α would have allowed to evaluate OLCs differentiation. The discrete area of analysis and the focal nature of the injury model are limitations to our study. Further investigation should include mild and diffuse injury models to improve the translatability to human cases of TBI. Also, microglia could have a critical role in gut microbial modulation of myelination, future studies should include in vivo microglia depletion and in vitro co-culture with OLs to address this mechanism.

In summary, depletion of the gut microbiota after TBI impaired OLC proliferation, increased OLC MHC-II expression, and reduced white matter remyelination. Absence of T-cells protected injured mice from the detrimental effects of gut microbial depletion on WM providing evidence for T-cells as a cellular mechanistic link for gut microbiota-brain communication in the setting of TBI. Future studies should address the molecular mechanisms of gut microbial regulation of the T-cell response (such as bacterial metabolites) and its role in regulating OLC differentiation and maturation in the setting of TBI.

### Electronic supplementary material

Below is the link to the electronic supplementary material.

Supplementary Material 1

Supplementary Material 2

Supplementary Material 3

## Data Availability

The datasets generated and analyzed during the current study are available from the corresponding author on reasonable request.
