# Supplementary material for: Early depletion of gut microbiota shape oligodendrocyte response after traumatic brain injury
Source: J Neuroinflammation. 2024 Jul 15;21:171. doi: 10.1186/s12974-024-03158-9 (PMC11251111; doi:10.1186/s12974-024-03158-9)
Supplement: Supplementary file 3 — Supplementary Material 3 [file 12974_2024_3158_MOESM3_ESM.docx]

| **Figure#** | **Variable** | **F statistic** | **P value** |
| --- | --- | --- | --- |
| 1.c | Injury/Sham | F(1,21) = 113.3 | < 0.0001 |
|  | VNAM/Kool-Aid | F(1,21) = 0.828 | 0.3732 |
|  | Interaction | F(1,21) = 16.29 | 0.0006 |
| 3.b | VNAM/Kool-Aid | F(1,15) = 0.003 | 0.9576 |
|  | T cell depletion | F(1,15) = 94.72 | < 0.0001 |
|  | Interaction | F(1,15) = 1.099 | 0.3113 |
| 3.c | VNAM/Kool-Aid | F(1,15) = 0.008 | 0.9266 |
|  | T cell depletion | F(1,15) = 45.77 | < 0.0001 |
|  | Interaction | F(1,15) = 0.1612 | 0.6941 |
| 3.f | VNAM/Kool-Aid | F(1,30) = 14.78 | 0.0006 |
|  | T cell depletion | F(1,30) = 9.97 | 0.0036 |
|  | Interaction | F(1,30) = 1.572 | 0.2195 |
| 3.g | VNAM/Kool-Aid | F(1,35) = 31.34 | < 0.0001 |
|  | T cell depletion | F(1,35) = 4.432 | 0.0425 |
|  | Interaction | F(1,35) = 7.986 | 0.0077 |
| 6.d | VNAM/Kool-Aid | F(1,18) = 5.241 | 0.0344 |
|  | Co-culture | F(1,18) = 118.9 | < 0.0001 |
|  | Interaction | F(1,18) = 7.903 | < 0.0116 |
| 6.e | VNAM/Kool-Aid | F(1,18) = 0.001 | 0.9738 |
|  | Co-culture | F(1,18) = 203.3 | < 0.0001 |
|  | Interaction | F(1,18) = 163.8 | < 0.001 |
| 6.f | VNAM/Kool-Aid | F(1,18) = 31.88 | <0.0001 |
|  | Co-culture | F(1,18) = 168.5 | < 0.0001 |
|  | Interaction | F(1,18) = 34.76 | < 0.001 |

**Supplemental Table 1. Two-way ANOVA F statistics.**
